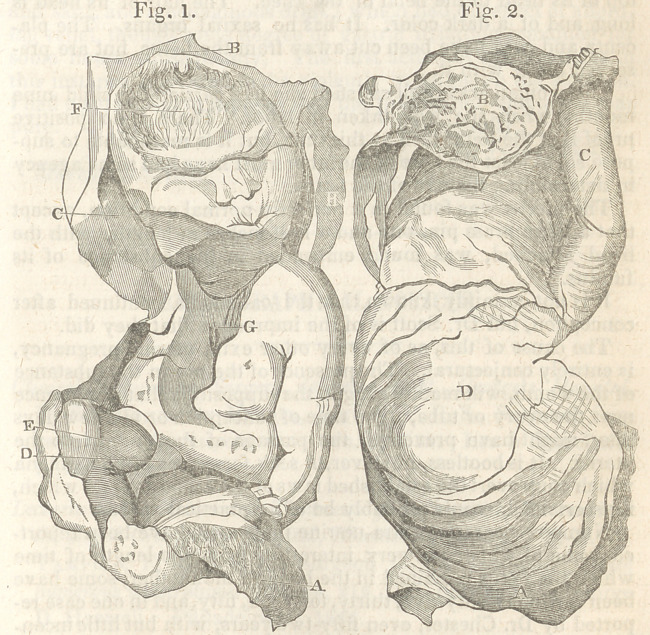# Case of Extra Uterine Pregnancy, in Which the Fœtus Was Carried Twenty-two Years

**Published:** 1846-05

**Authors:** John W. Craddock

**Affiliations:** Halifax County, Va.


*Case of Extra Uterine Pregnancy, in which the Fœtus was carried twenty-two years.* By JOHN W. CRADDOCK, M. D., of Halifax County, Va.

The subject of this pregnancy was a mulatto woman, the property of Mr. Charles Walker, of Campbell County, Virginia, in whose possession she remained until her death, which took place twenty-two years after she became pregnant, and in the forty-fourth year of her age. It was her first and only pregnancy, and happened about the age of twenty-one. Up to the usual period of utero-gestation, nothing remarkable occurred to her. At this period she was seized with labor-pains of the usual character, which continued at intervals for three days, without any result as far as the birth of the child was concerned. During this period, she had the attention of several respectable and accomplished physicians, among whom was Dr. Raleigh White, Sr., a practitioner of much celebrity, and well known in Virginia. This gentleman concurred in the opinion that the case was one of extra-uterine pregnancy, and as her sufferings ceased at the expiration of the above mentioned term, advised that there should be no interference with the course of nature.

She was accordingly abandoned by her medical attendants, and in a short time resumed her labors as a cook and washer-woman; in which capacity she served for twenty-one years, not in good health, but never confined by illness, and always presenting the appearance of an advanced stage of gestation. At the expiration of the above time, she was attacked with an acute pneumonia, which terminated her life after a short illness, without any uncommon symptom.

After death, Dr. Scott made a careful examination of the thoracic and abdominal cavities, and extracted the fœtus with its appendages and cyst. Dr. S. found the cyst occupying the greater basin and superior strait of the pelvis, to be of a triangular form, nine inches long, by seven and a half at its greatest breadth. The small intestines were entirely displaced, but the uterus and bladder appeared to retain their natural relations to the sides of the pelvis. The attachments of the cyst consisted first of a cord three-fourths of an inch in diameter, containing many blood vessels, passing from its lower extremity into the space between the bladder and uterus, where it was firmly fixed, and secondly of a cord three inches in length, and some two lines in thickness, passing between the other extremity of the cyst and the omentum major.

Upon removing the cyst in order to examine its contents, the parietes were found to be ossified to so great an extent as almost to resist the scalpel, the ossification being very general and involving also the placenta, which was fixed on the inside of the cyst, at its lower extremity; this ossification of the membranes displays well the process as it occurs in the flat bones of the body. The fœtus, as will be seen in the annexed cut, held a position not unfrequently met with under natural circumstances, except that the long continued pressure exerted upon it had, probably by causing an absorbtion of the waters, condensed it by pressing the parts out of their natural form.

Fig. 1.

Fig. 2.

Fig. 1. *Front view.*

A. Lower portion of divided placenta. B. Upper portion of do. turned back from the head. C. Right arm raised by the side of the head. D. Umbilical cord compressed into an angular shape, and passing downwards, between the legs to reach the back part of the placenta A. E. Right thigh and knee, corresponding to the indentation in the forehead. F. In the position in which they were found in the cyst. G. Depression in the breast caused by the compression of the chin. H. Fragments of the ossified membranes of the cyst.

Fig. 2. *Back view.*

A. Lower portion of the divided placenta.

B. Upper part of do. turned back, showing the ossific and fibrous structure.

C. Right arm by the side of the head.

D. Convexity of the cyst.

The fœtus is fully formed, and measures nineteen inches in its longest diameter, and sixteen in the short, and thirteen from the top of its head to the bend of the knee. The hair of its head is long and of a dark color. It has no sexual organs. The placenta and cord have been cut away from the fœtus, but are preserved along with it.

The woman was well satisfied she had carried the child nine months, when she was taken in labor. There is no positive proof that the fœtus died at this time, but it is reasonable to suppose so, at least the time of the labor must have had some agency in determining the age of the fœtus.

The uterus was found in a perfectly normal condition, except that a large brass pin, one and a half inches in length, with the head attached, was found embedded in the substance of its fundus.

It is not certainly known that the catamenia continued after conception, but Dr. Scott is of the impression that they did.

The cause of this, as of every other extra-uterine pregnancy, is entirely conjectural. The presence of the pin in the substance of the womb, will readily suggest the supposition that its presence near the ovary or tube, at the time of conception or in a few days after, might have prevented the passage of the ovum into the uterus. It is bootless, however, to seek for causes of phenomena which controvert the established laws of the economy, and which, if ascertained, would probably be of no practical value.

Numerous cases of extra-uterine pregnancy have been reported, some of which are very interesting from the length of time which the fœtus remained in the body of the mother; some have been carried twenty-five, thirty, forty-six, fifty, and in one case reported by Dr. Chester, even fifty-two years, with but little inconvenience to the mother, except from the sensation of weight.

## Figures and Tables

**Fig. 1. Fig. 2. f1:**